# Risk-Reduction Strategies for Platelet Transfusion in the United States

**DOI:** 10.1100/tsw.2011.60

**Published:** 2011-03-07

**Authors:** Eleftherios C. Vamvakas

**Affiliations:** Department of Pathology and Laboratory Medicine, Cedars-Sinai Medical Center, Los Angeles, CA, USA

**Keywords:** transfusion-transmitted infections, post-transfusion bacteremia/sepsis, pathogen reduction, pathogen inactivation, Intercept, Mirasol®, apheresis platelets, single-donor platelets, platelet transfusion, bleeding in hemato-oncology patients, multicomponent apheresis, transfusion-related acute lung injury (TRALI

## Abstract

Despite bacterial culture of platelets, transfusion-associated bacteremia/sepsis (TABS) may occur with a frequency of approximately 1/60,000 platelet transfusions, while an emerging transfusion-transmitted infection (TTI) could reproduce the epidemic of transfusion-transmitted human immunodeficiency virus (HIV) in the future. As platelet pathogen-reduction (PR) systems licensed in Europe may eventually become licensed in the U.S., three alternative strategies for reducing the residual risks of TTIs and TABS may become available in the U.S. in the future: (1) transfusion of (already-available) non–pathogen-reduced single-donor (as opposed to pooled whole-blood-derived [PWBD]) platelets, (2) transfusion of pathogen-reduced single-donor platelets, or (3) transfusion of pathogen-reduced PWBD platelets (if trials of this component are conducted in the U.S. in the future). PR of platelets will increase the risk of mild and moderate (albeit perhaps not severe) bleeding complications and it cannot protect from all pathogens. Compared to PWBD platelets, single-donor platelets can reduce, by at least twofold, the risk of all known and emerging TTIs, as well as the risk of TABS, without incurring any risk. The fewer donor exposures secured by the use of single-donor platelets – especially if combined with collection of red blood cells and/or plasma from the same donation for transfusion to the same recipient through the use of multicomponent apheresis – may also reduce the risk of transfusion-related acute lung injury. To choose between pathogen-reduced and non–pathogen-reduced single-donor platelets, the increased risks of bleeding complications as well as other possible adverse events secondary to PR need to be quantified precisely and weighed against the competing risks of TABS and emerging TTIs.

## INTRODUCTION

In the U.S., bacterial culture of single-donor platelets was mandated by the AABB (formerly American Association of Blood Banks) in 2004, but the risk of transfusion-associated bacteremia/sepsis (TABS) is still estimated at 1/60,000 single-donor platelet transfusions owing to the reduced clinical sensitivity of the bacterial culture methods used[1]. As of January 31, 2011, the AABB also mandated bacterial culture of pooled whole-blood-derived (PWBD) platelets for its institutional members[2,3]; the U.S. Food and Drug Administration (FDA) has not set a requirement for bacterial detection in platelet components. When bacterial culture methods of equivalent sensitivity are used simultaneously for both PWBD platelets manufactured by the U.S. platelet-rich-plasma (PRP) method and single-donor platelets, PWBD platelets are associated with a 5.6-fold ($p < 0.05$) higher risk of bacterial contamination compared with single-donor platelets[4]. This is because the source of the bacteria causing TABS is usually the donor's skin and PWBD platelets entail four to six donor venipunctures to procure one therapeutic platelet dose that could have been harvested instead by pheresing one plateletapheresis donor[5,6].

Furthermore, each blood donor contributing to the procurement of one WBD platelet dose can potentially be the source of a transfusion-transmitted infection (TTI)[1,4]. Accordingly, PWBD platelets have at least a twofold higher risk of TTIs compared with single-donor platelets[4]. Although the risk of known TTIs in the U.S. is exceedingly low[7], emerging TTIs can potentially reproduce the epidemic of transfusion-transmitted human immunodeficiency virus (HIV) infection of the early 1980s or the epidemic of transfusion-transmitted West Nile Virus (WNV) infection of the summer and fall of 2002[4]. The AABB prioritized 68 potential or actual transfusion-transmitted agents based on both the existing evidence of their transmissibility by transfusion and their potential for severe clinical outcomes[8]. The highest-priority agents were the variant Creutzfeldt-Jakob disease (vCJD) prions, dengue-fever virus, and *Babesia* sp.[8]. Additional emerging transfusion-transmitted agents of significant concern (such as the xenotropic murine leukemia-related virus [XMRV][9]) have continued to be reported after the publication of the AABB compendium[8].

When bacterial culture of platelets was initially mandated by the AABB for its institutional members, bacterial culture methods were available only for single-donor platelets. For this reason, AABB-accredited U.S. blood establishments moved voluntarily toward reliance on single-donor platelets so that 87.5% of platelet doses transfused in the U.S. in 2006 were single-donor platelets[10]. In addition, the platelet pathogen-reduction (PR) systems[11,12,13] Intercept and Mirasol®, which use chemicals (respectively, amotosalen HCl along with ultraviolet-A light or riboflavin along with ultraviolet-B light) to inactivate nucleic acids in platelet concentrates, were licensed in Europe for the purpose of confronting the residual risk of TABS and TTIs. Thus, to mitigate the residual risks of TABS and TTIs from platelet transfusion, three alternative risk-reduction strategies may eventually reach the U.S. market: (1) transfusion of already-available non–pathogen-reduced single-donor (as opposed to PWBD) platelets, (2) transfusion of pathogen-reduced single-donor platelets, or (3) transfusion of pathogen-reduced PWBD platelets (if trials of this component are conducted in the U.S. in the future).

If the available PR systems resulted in unacceptable increases in bleeding complications *and* no available PR system was capable of inactivating all emerging pathogens, we should logically rely on non–pathogen-reduced single-donor platelets. Alternatively, if PR could be achieved with a tolerable increase in bleeding complications and trials of pathogen-reduced PWBD platelets conducted in the U.S. yielded satisfactory results, we could perhaps rely on pathogen-reduced PWBD platelets. However, if the reduction in the number of allogeneic-donor exposures (from four to six down to one) also conferred other (noninfectious) benefits, *or* if no PR system was capable of inactivating all pathogens, then — provided that PR could be achieved with a tolerable increase in bleeding complications — we should rely on pathogen-reducing single-donor (as opposed to PWBD) platelets.

Now that consideration of the totality of the evidence on the bleeding complications of pathogen-reduced platelets is possible, following the publication of three manufacturer-sponsored studies and an independently funded randomized controlled trial (RCT) of the Intercept technology[14], as well as the publication of the manufacturer-sponsored RCT of the Mirasol technology[15], this review will update the previous review[4] on the choice between PWBD and single-donor platelets. Because all three aforementioned risk-reduction strategies may eventually become available in the U.S., this review will examine the considerations pertinent to the selection between these three strategies. For this purpose, the PudMed search of the previous review[4] will be extended to June 30, 2010, along with the literature search of a recent meta-analysis[16] of the RCTs comparing the frequency of bleeding complications between recipients of non–pathogen-reduced vs. pathogen-reduced platelets. The following will be considered: (1) the *incremental* risk of TTIs and TABS secondary to PWBD (compared with single-donor) platelets, (2) the risk of bleeding complications secondary to PR, (3) whether PR can protect from all pathogens, and (4) the possibility of other (noninfectious) benefits from the reduction in allogeneic-donor exposures thanks to the transfusion of single-donor (rather than PWBD) platelets.

## WHAT IS THE *INCREMENTAL* RISK OF TTIs AND TABS SECONDARY TO THE PWBD PLATELETS STILL TRANSFUSED IN THE U.S.?

If the prevalence of the next TTI to emerge in blood donors were p, we could assume that (1) a particular transfusion recipient has a negligible probability of receiving more than one infectious unit (i.e., each distributed blood component contaminated with the new pathogen will infect a different transfusion recipient and will therefore represent a new case of the TTI); (2) all transfusion recipients are at risk, that is, there is a negligible probability of an infectious component being given to a transfusion recipient who is already infected; and (3) the probability of at least one of the four, five, or six donors in a pool of four to six WBD platelets being infectious (i.e., $1-[1-p]^4$ to $1-[1-p]^6$, respectively) approximates 4p to 6p.

In the case of an emerging transfusion-transmitted virus similar to HIV or WNV, infectivity in blood or plasma (number of infectious copies per milliliter) is usually so high that any patient transfused with even a few milliliters of blood or plasma could contract infection. Thus, if the next TTI to emerge had the characteristics of (chronic) HIV or (acute) WNV infection, pools of four or six platelet concentrates would have four or six times the transmission risk associated with a single-donor platelet concentrate, no matter what manipulations were attempted during the manufacturing process to minimize the plasma contribution of each of the four to six individual donors to the platelet pool. (The latter manipulations usually consist of the removal of as much of the plasma from each platelet concentrate as possible and the resuspension of the platelets in a platelet-additive solution.)

Although the fact that a pooled product is associated with a higher risk of TTIs than a single-donor product may appear obvious, the precise magnitude of the increase in risk in association with a PWBD platelet pool compared with a single-donor concentrate is hard to estimate because competing assumptions (Table 1) enter into the calculation. The two ends of the plausible range (i.e., the “best-case” vs. the “worst-case” scenario) are shown in Table 1 as a twofold vs. a sixfold difference in risk[4]. More specifically, if the pools are made from four donors, no recipient of a platelet dose receives a second or third platelet dose owing to an inadequate rise in post-transfusion platelet count following the first dose and *all* single-donor platelet concentrates are split; pools will have a two times higher risk of transmitting TTIs compared with single-donor platelets. Alternatively, if the pools are made from six donors, twice as many platelet doses are given to the entire population of platelet transfusion recipients owing to inadequate rises in post-transfusion platelet count in some of the patients and (as in the aforementioned best-case scenario) *all* single-donor platelet concentrates are split; pools will have a six times higher risk of transmitting TTIs compared with single-donor platelets.

Given the assumptions made under the “best-case” scenario, the plausible difference in risk *cannot* be less than a twofold increase in the risk of TTIs from transfusion of PWBD (rather than single-donor) platelets. However, the upper end of the plausible range could be higher than a sixfold increase in risk if (1) more than twice as many platelet doses were given to the entire population of platelet transfusion recipients owing to inadequate post-transfusion corrected count increments (CCIs) in some patients and/or (2) not all apheresis components collected by U.S. blood establishments were split. Because the choice

**TABLE 1**
**Competing Assumptions Leading to the Calculation of the Two Ends of the Plausible Range for the Increase in the Risk of TTIs Secondary to Transfusion of PWBD (Rather than Single-Donor) Platelets**

| "Best-Case" Scenario: *Twofold* Increase in Risk* | "Worst-Case" Scenario: *Sixfold* Increase in Risk** |
|---|---|
| • Pools consist of four WBD platelet concentrates | • Pools consist of six WBD platelet concentrates |
| • No recipient of a platelet dose receives a second or third platelet dose owing to inadequate post-transfusion CCIs | • Because dose escalation is the usual approach to nonimmune causes of refractoriness to platelet transfusions, twice as many platelet doses are given to the entire population of platelet transfusion recipients owing to inadequate post-transfusion CCIs in some patients |
| • 100%*** of single-donor platelet concentrates are split (so that an infected plateletapheresis donor can infect two [or even three] transfusion recipients) | • 100% of single-donor platelet concentrates are split† |

* (4 × 1)/2 (respectively for each of the three assumptions) equals 2.

** (6 × 2)/2 (respectively for each of the three assumptions) equals 6.

*** 64% of single-donor platelet concentrates collected in the U.S. in 2006 were split[10].

† In theory, the "worst-case" scenario would pertain to a situation in which 0% of single-donor platelet concentrates are split. In this case, the "worst-case" scenario would correspond to a 12-fold increase in risk (i.e., taking into account only the first and second assumption in this column, 6 × 2 = 12). Although this would indeed be the "worst-case" scenario in theory, the table depicts the "worst-case" scenario in practice: what may be the worst version of the current *reality*, given the manufacturing practices of U.S. blood centers[10].

between the risk-reduction strategies considered in this review depends only on the lower end of the plausible range, the estimation of the upper end of the range will not be pursued further.

The prediction that recipients of PWBD platelets will be exposed, through platelets, to *at least* twice as many allogeneic donors compared with recipients of single-donor platelets has been corroborated by empirical observations. A recent study[17] that used pools consisting of four WBD platelets considered the *total* number of allogeneic donors (including donors of red-blood-cell [RBC] and plasma components) to whom recipients of PWBD vs. single-donor platelets had been exposed. Compared with recipients of single-donor platelets, hemato-oncology patients receiving PWBD platelets were exposed to 2.6–2.8 times as many, and surgical patients to 1.7–1.9 times as many, *total* allogeneic donors. Similarly, when pools of seven WBD platelets were used from 1991–1995[18], the number of *total* allogeneic donors to whom each platelet transfusion recipient was exposed in association with PWBD (compared with single-donor) platelets was 3–4 times higher for hemato-oncology and 1.5–2 times higher for cardiac-surgery patients.

The plausible range shown in Table 1 presumes that two other assumptions cancel each other out. The first of these assumptions pertains to the potentially lower risk of known TTIs in plateletapheresis (compared with whole-blood) donors. The second assumption concerns the possibility that infected plateletapheresis donors may donate more than once during the window period of hepatitis B virus (HBV) infection.

The prevalence of HIV, hepatitis C virus (HCV), and HBV infection is six to 25 times ($p < 0.05$) lower in American Red Cross (ARC) plateletapheresis (compared with whole-blood) donations[19]. Such a lower TTI prevalence would translate into a lower risk of transmission of these TTIs after transfusion of single-donor compared with PWBD platelets if the lower TTI prevalence correlated with lower TTI incidence in donors. This author knows of no published evidence of such a correlation[20]. When HIV-nucleic-acid, HCV-nucleic-acid, and HBc (antibody to the HBV core antigen) testing is performed in addition to testing for HIV and HCV antibody and PRISM HBsAg (HBV surface antigen), the window period of HIV, HCV, and HBV infection (in days, along with the associated 95% confidence interval [CI]), is 9.5 (8.2–10.8), 8.0 (6.8–9.2), and 38.3 (33.0–43.7) days, respectively[21]. It is possible that a donor who has denied all risk factors may develop incident HBV infection during the latter 38.3-day window period; cases of such multiple transmissions (by an infected plateletapheresis donor who donates more than once during the window period of her/his HBV infection) have been reported[22], albeit only in settings not testing for HBc. This author knows of no such case reported from a blood system testing for HBc.

Even in countries testing for HBc, however, the possibility remains of a plateletapheresis donor donating more than once during the window period of HBV infection, as does the possibility that a 25-fold lower prevalence of HBV infection in plateletapheresis (compared with whole-blood) donors[19] may correlate with a lower incidence of HBV infection in donors denying all risk factors and, therefore, with a lower risk of HBV transmission to platelet transfusion recipients. As there is no published evidence for either of these possibilities, these possibilities were not included in the calculation of the two- to sixfold plausible range for the difference in the risk of TTIs between PWBD vs. single-donor platelets shown in Table 1.

Fig. 1 shows the difference in the risk of bacterial contamination of PWBD vs. single-donor platelets across U.S. studies[23,24,25] that simultaneously performed bacterial culture on PRP pools and plateletapheresis components under the same conditions[4]. Because the starting concentration of bacteria from a donor's skin contaminating a platelet component during venipuncture is exceedingly low, it is possible that *no* bacteria may be present in the ≤10 mL of sample inoculated into the culture media. Accordingly, despite the use of culture methods with excellent *analytical* sensitivity, the *clinical* sensitivity of the bacterial culture of platelet components is only ≤50%[26,27]. The clinical sensitivity of culture methods can be increased by taking a sample for bacterial culture after a platelet concentrate has been stored for ≥36 h at room temperature (so that bacteria have been allowed to proliferate during storage), by taking a sample for bacterial culture before the issuance of the product for transfusion, or by combining the initial culture with additional point-of-release testing[28].

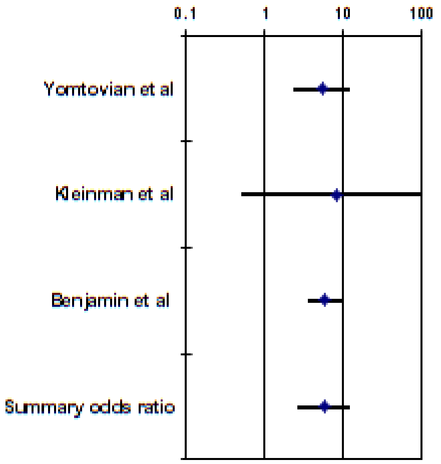

**FIGURE 1.** True bacterial contamination of PWBD platelets manufactured by the PRP method in the U.S. vs. single-donor platelets cultured simultaneously under the same conditions. The figure shows the results of the three available studies[23,24,25] as the summary OR of bacterial contamination in PRP PWBD vs. single-donor platelets across the three studies calculated by a meta-analysis[4]. There is complete concordance between the available studies[23,24,25], with a >97.5% probability that the observed variation in their results could have arisen by chance ($p > 0.975$ for the Q test statistic)[4]. Across the studies[23,24,25], pools of platelet concentrates made from five donors are associated with a 5.6-fold increase in the risk of contamination (summary OR, 5.58; 95% CI, 2.6–12.0; $p < 0.05$). The difference in bacterial contamination between PWBD and single-donor platelets was not observed in the same meta-analysis[4] across the European studies of PWBD platelets manufactured by the buffy-coat method.

The same reasoning for the plausible range of the difference in risk (presented in Table 1 for TTIs) applies to TABS. If pools of five WBD concentrates have a 5.6 times higher risk of bacterial *contamination* than single-donor platelets (summary odds ratio [OR], 5.6; 95% CI, 2.6–12.0; i.e., including the expected value of 5 from the five venipunctures), then — based on the competing assumptions shown in Table 1 — PWBD platelets will have at least a twofold higher risk of TABS compared with plateletapheresis components.

Platelets were prepared from 16.9% of allogeneic whole-blood units in 2006 compared with 30.3% in 2004[10]. An even larger reduction in the proportion of platelet pools had been realized between 2002 and 2004, indicating that — had there been a mandate to do so — blood establishments could probably have replaced the remaining pools with single-donor platelets by 2009. Because of dissemination of information concerning a (purportedly) reduced risk of transfusion-related acute lung injury (TRALI) secondary to transfusion of PWBD (rather than single-donor) platelets, however, in August-September 2009, 22% of U.S. blood centers responding to a questionnaire from an AABB-appointed TRALI Working Group stated that they endeavored to increase production of PWBD platelets in an effort to mitigate the risk of TRALI from single-donor platelets. Eight of 10 blood centers proffering this response as a TRALI risk-reduction strategy were increasing production of PWBD platelets regardless of the gender of the donors contributing to a platelet pool[29]. Accordingly, when the 2008 U.S. transfusion data are released, a smaller proportion of platelet doses may have been transfused as single-donor concentrates compared with the 87.5%[10] of platelet doses transfused as single-donor concentrates in 2006.

Of 10.388 million WBD-equivalent platelet units transfused in the U.S. in 2006, 1.296 million (12.5%) were transfused as pools[10]. Rather than relying on 1.296 million whole-blood donors, these platelet doses could have been collected from 216,000 plateletapheresis donors, thereby avoiding 1.080 million exposures of platelet transfusion recipients to allogeneic donors. The risk from continuing to transfuse 12.5% of platelet doses as pooled platelets is the annual number of infectious platelet pools that would no longer be transfused in the U.S. if all platelet doses were transfused as single-donor platelets. These *preventable* donor exposures from the continued use of pools result in a small number of HIV, HCV, and HBV transmissions and 15 cases of TABS every year (Fig. 2). Equally importantly, these *preventable* donor exposures would generate 36.1–108.2 transmissions annually if the next major transfusion-transmitted agent to emerge in the U.S. were “HIV-like”, and 9.9–29.7 transmissions annually if the next agent were “WNV-like” (Fig. 2).

More specifically, if the next agent produced chronic infection like HIV, the 1.080 million preventable donor exposures would cause at least 36.1 preventable transmissions in the year preceding the implementation of testing (assuming that the new agent will be detected a lot faster than HIV was detected: when its prevalence in blood donors is 10 times less than the 1984 HIV prevalence, i.e., 1/10,000 [as opposed to 1/1,000] donors). If the next agent transmitted infection only during that short period of acute viremia and symptomatic illness (like WNV), the preventable donor exposures would cause at least 9.9 preventable transmissions during its first epidemic if the events of 2002 (when WNV caused an estimated 380 transfusion transmissions[30]) were repeated[4].

## CAN PR BE ACHIEVED WITH A TOLERABLE INCREASE IN BLEEDING COMPLICATIONS?

Five RCTs[14,15,31,32,33] studied the bleeding complications of PR in patients receiving routine transfusions[16]. Transfusions were “routine” if the decision to transfuse was made by a patient’s own physician and if the components were not manipulated in ways other than PR[16]. Across the studies[14,15,31,32,33], compared with patients receiving non–pathogen-reduced platelets, subjects receiving pathogen-reduced platelets had a significant ($p < 0.05$) 58% increase in all bleeding complications (summary OR, 1.58; 95% CI, 1.11–2.26) and a significant 54% increase in clinically significant bleeding complications (summary OR, 1.54; 95% CI, 1.11–2.13)[16]. The frequency of severe

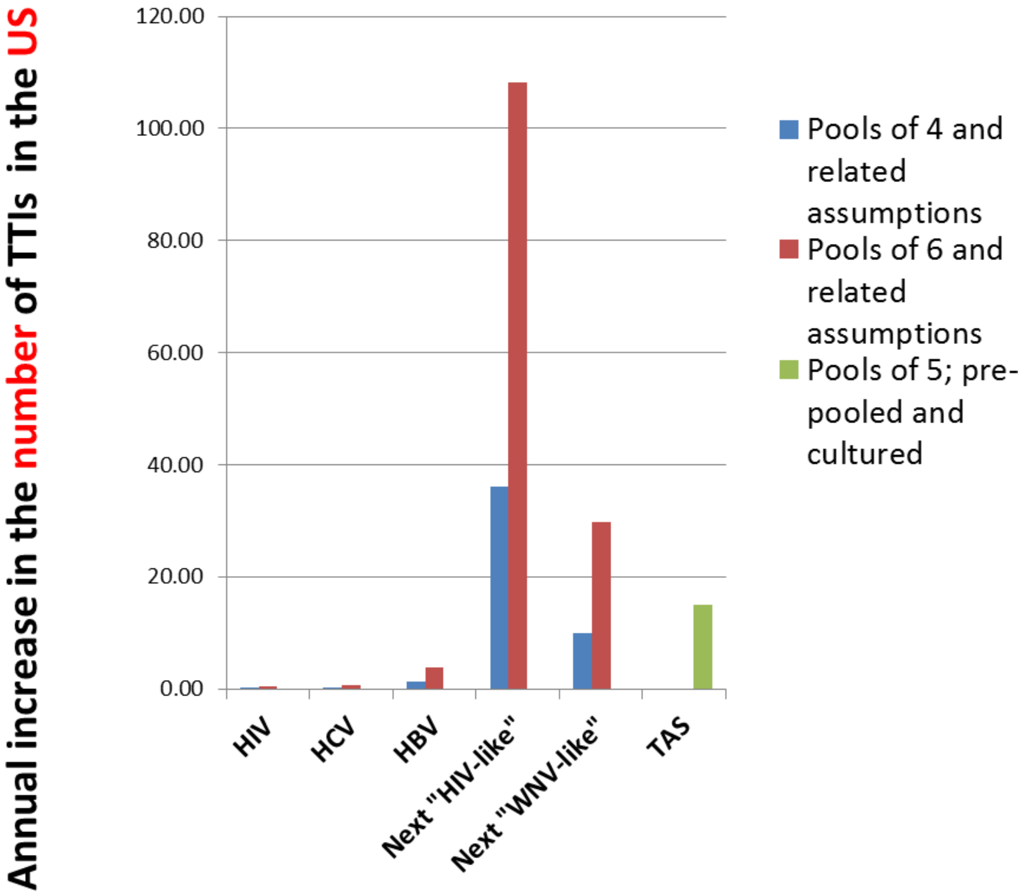

**FIGURE 2.** Annual increase in the number of TTIs contracted in the U.S. because of the 1.080 million preventable allogeneic-donor exposures that result from the continued transfusion of 12.5% of platelet doses as pooled (rather than single-donor) platelets[10]. Because competing assumptions were involved in the calculations (Table 1), each pair of columns for the HIV, HCV, or HBV infection, as well as infection with the next "HIV-like" or "WNV-like" (see text) transfusion-transmitted pathogen to emerge in the U.S., corresponds to the two ends of the plausible range[4]. The preventable transmissions of HIV, HCV, or HBV infection are due to "window-period" infections. Observed numbers of known TTIs do not approach these modeled estimates, either because transmissions are under-recognized or under-reported with current surveillance, or because the assumptions made in the published models[7] are overly conservative. The depicted annual risk of TABS reflects the situation in the U.S. as of January 31, 2011 when bacterial culture of PWBD platelets was implemented. Abbreviations used in the figure: HIV = human immunodeficiency virus; HCV = hepatitis C virus; HBV = hepatitis B virus; TAS = transfusion-associated bacteremia/sepsis; WNV = West Nile Virus.

bleeding complications did not differ (summary OR, 1.25; 95% CI, 0.86–1.81), although the 1,080 subjects studied[14,15,31,32,33] may be too few for establishing a difference of this magnitude[16].

Severe bleeding was bleeding categorized as such by the investigators, or equal to or greater than grade 3 on the CTC (Common Toxicity Criteria of the National Cancer Institute) scale[34], or, if results on the CTC scale had not been reported, bleeding equal to or greater than grade 3 on the WHO (World Health Organization) scale[35]. Clinically significant bleeding was bleeding equal to or greater than grade 2 on the CTC or WHO scale. Briefly, grade 1 bleeding comprises minimal bleeding not requiring intervention. Grade 2 bleeding comprises symptomatic bleeding for which minimal intervention (e.g., steroid suppositories for rectal bleeding or ≥2 pads per day for vaginal bleeding) is required. Grade 3 bleeding requires transfusion or other intervention. Grade 4 bleeding is catastrophic. Distinction between grade 1 and grade 2 bleeding is subtle[36]. Grading bleeding is difficult and observers often disagree, especially when it comes to distinguishing between grades 1 and 2. Thus, for all bleeding and for severe bleeding, reliable data could be retrieved from all five RCTs[14,15,31,32,33]. For "clinically significant" bleeding, data could be extracted from only three studies[14,15,37], and were less reliable than the data on all and on severe bleeding.

Only two studies, however, had assessed bleeding *daily*, using trained assessors, whether blinded assessors in the SPRINT trial[32,37] or unblinded assessors in the HOVON trial of Kerkhoffs et al.[14]. When these two studies are integrated in a meta-analysis, PR is associated with a significant ($p < 0.05$) increase in all clinically significant as well as severe bleeding complications (Fig. 3). More importantly, whether the analysis includes all studies[14,15,31,32,33] or is limited to the latter two studies[14,32,37], the variation in the findings of the studies is sufficiently modest to have arisen by chance[16].

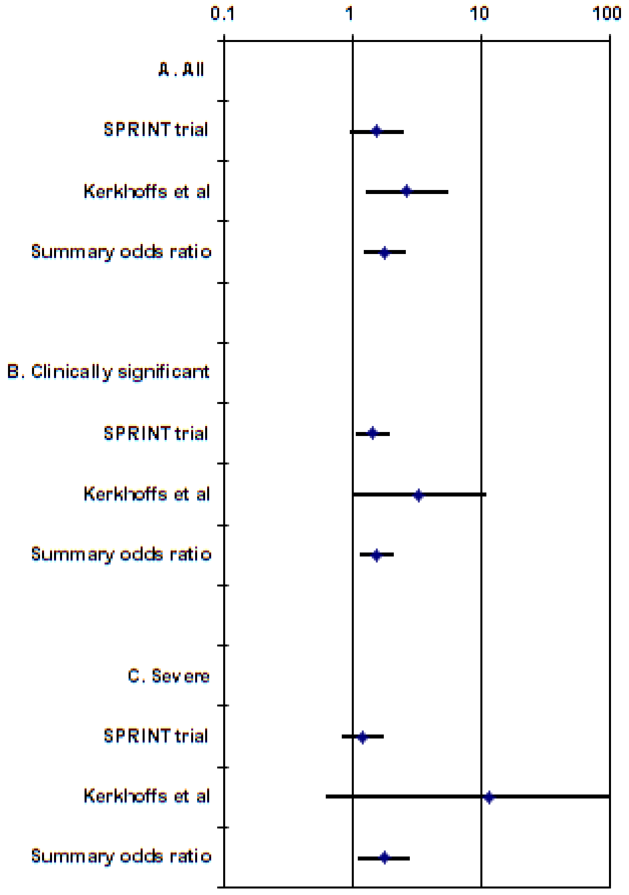

**FIGURE 3.** ORs of all bleeding complications, clinically significant bleeding complications, and severe bleeding complications in patients receiving pathogen-reduced vs. non–pathogen-reduced platelets in the two RCTs that assessed bleeding daily using trained assessors[14,37]. The bleeding complications recorded in the entire study period (including both the transfusion and surveillance period) of the SPRINT RCT[32] were reported separately[37]. The figure also shows the summary OR (95% CI) of bleeding complications across the two RCTs[14,37], which is statistically significant ($p < 0.05$) in all three analyses: 1.8 (1.2–2.7) for all bleeding complications, 1.5 (1.1–2.1) for clinically significant bleeding complications, and 1.8 (1.1–2.8) for severe bleeding complications.

The findings of the four studies of the amotosalen-HCl/ultraviolet-A-light (Intercept) technology[14,31,32,33] correlate with the kind of population of hemato-oncology patients enrolled in each study. More specifically, studies that enrolled *selected* hemato-oncology patients[31,33] found no difference in bleeding between recipients of pathogen-reduced vs. non–pathogen-reduced platelets; the SPRINT trial[32,37], which enrolled moderately selected hemato-oncology patients, found a difference in clinically significant[37] (but not in all) bleeding complications between recipients of pathogen-reduced vs. non–pathogen-reduced platelets; and the recent, independently funded trial of Kerkhoffs et al.[14], which enrolled *unselected* hemato-oncology patients, found a much more pronounced difference in (all and clinically significant) bleeding between its randomization arms than had been observed in the SPRINT trial. Patients were "selected" if subjects with splenomegaly, disseminated intravascular coagulation (DIC), and/or fever, in addition to other factors predisposing to platelet consumption, were excluded[31,33]. Patients were "moderately selected" if DIC and splenomegaly were not listed among the exclusion criteria[32]. Patients were "unselected" if only patients with HLA antibodies requiring support from HLA-matched donors, in addition to pregnant or pediatric patients, were excluded[14].

In the future, the experience from all available RCTs[14,15,31,32,33] should always be considered in its entirety. Only when all 1,080 subjects[14,15,31,32,33] are considered together is the entire population of hemato-oncology patients represented and the reported results represent a *continuum*, so that the recent findings of Kerkhoffs et al.[14] are not inconsistent with the earlier literature[16].

Although PR results in a predictable loss of platelets, the cellular losses do not exceed one-third of the total platelets[38] and should not have produced the threefold increase in clinically significant bleeding (Fig. 3) recorded in the trial of Kerkhoffs et al.[14]. Since the patients of Kerkhoffs et al.[14] received prophylactic platelet transfusions when their platelet count fell below 10,000 μL, and the PLADO (Platelet Dose) RCT showed that recipients of half-the-standard platelet dose have the same risk of bleeding as recipients of the standard (as well as double-the-standard) platelet dose[39], Kerkhoffs et al.[14] would have been expected to find no increase in bleeding complications owing to the platelet losses induced by PR. If the expected platelet losses[38] cannot account for the excess bleeding observed with PR[14], perhaps PR causes functional impairment of all the pathogen-reduced platelets in addition to causing loss of viability of a proportion of the treated platelets. Functional impairment of all the treated platelets could be expected from the damage to the mitochondrial DNA induced by PR[40,41]. If this is the case, contrary to what we had previously assumed[1,11,12,13], the platelet damage from PR cannot be overcome by merely increasing the platelet dose[14,16]. Introduction of PR in its current stage of development will result in an increase in mild and moderate, albeit perhaps not severe, bleeding complications, which we will have to explicitly tolerate to reap the benefits[1,11,12,13] conferred by PR.

The study of the riboflavin/ultraviolet-B-light (Mirasol) technology[15] accounted upfront for the platelet losses inflicted by PR by not transfusing the pathogen-reduced components in which the platelet content was below the minimum allowed by French standards ($3.0 \times 10^{11}$ platelets per transfused component). Despite this, the study of the Mirasol technology[15] failed to show noninferiority of the pathogen-reduced platelets. What the transfusion-medicine community is willing to tolerate to reap the benefits of PR was *a priori* stated to be a 20% reduction in the 1-h post-transfusion CCI. However, the mean difference in the 1-h CCI between recipients of treated and untreated platelets was 5,214 and the 95% CI of the mean difference (2,887–7,452) contained the *a priori* specified upper limit (2,940) of the zone of noninferiority. Although a subsequent study of the Intercept technology (hitherto reported only in abstract form) found noninferiority of pathogen-reduced platelets stored past the allowed storage time, this study[42] had defined noninferiority as a "tolerable" 30% reduction in the 1-h post-transfusion CCI.

Fig. 4 shows the mean difference in the 1- and 24-h CCIs between recipients of non–pathogen-reduced and pathogen-reduced platelets in the five available RCTs[14,15,31,32,33]. In all cases — the Mirasol trial[15], the SPRINT trial[32], and across the three other studies[14,31,33] — there is a significant ($p < 0.05$) difference favoring the control arm.

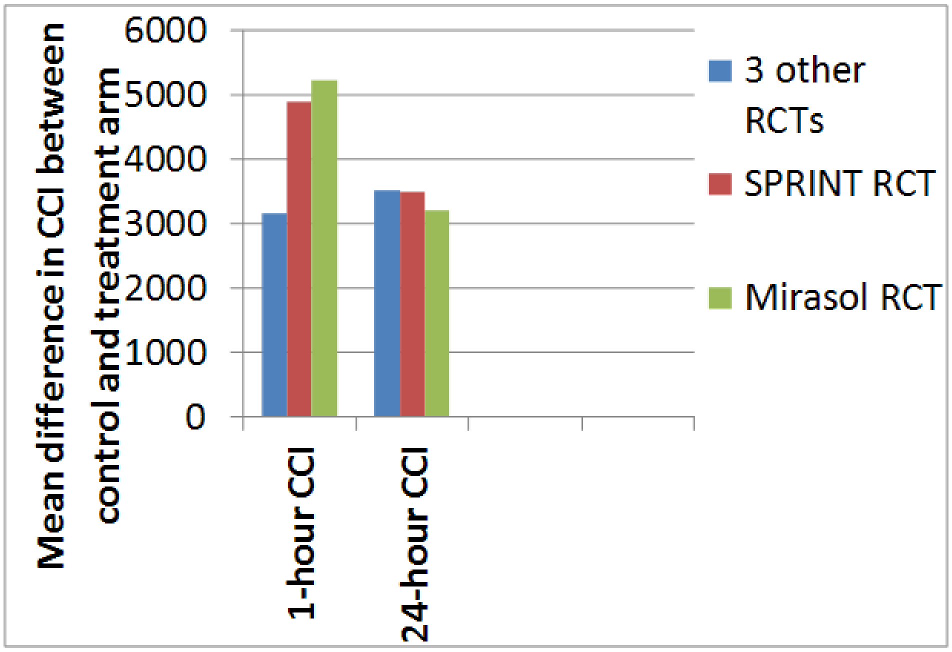

**FIGURE 4.** Mean difference in the 1- and 24-h CCIs between recipients of non–pathogen-reduced vs. pathogen-reduced platelets in the study of the Mirasol technology[15], the SPRINT RCT of the Intercept technology[32], and across the three other RCTs[14,31,33] of the Intercept technology. Because the SPRINT trial[32] had not reported the SD of the difference for these quantitative outcomes, it could not be integrated with the other RCTs in the meta-analysis[16]. All depicted analyses demonstrate a statistically significant ($p < 0.05$) reduction in CCIs secondary to PR.

The SPRINT RCT[37] reported serious adverse events in 27% (86/318) of the recipients of pathogen-reduced platelets and in 25% (81/327) of the recipients of non–pathogen-reduced platelets ($p = 0.53$). Although the differences between the arms in nearly all individual categories of adverse events were not significant, there were significant ($p < 0.05$) differences in the risk of pleuritic pain, pneumonitis (not otherwise specified), and acute respiratory distress syndrome (ARDS), all of which had occurred more frequently in recipients of pathogen-reduced (compared with non–pathogen-reduced) platelets. ARDS had occurred in 1.6% of recipients of pathogen-reduced platelets compared to 0% of controls ($p = 0.03$); pneumonitis had occurred in 2.2% of recipients of pathogen-reduced platelets compared to 0% of controls ($p < 0.01$).

Additional investigation revealed that there had been inconsistent use of the terms pneumonitis and ARDS among study sites in the reporting of adverse events[37]. A reanalysis of retrospectively collected pulmonary data on 148 patients (78 recipients of pathogen-reduced platelets and 70 recipients of non–pathogen-reduced platelets, all of whom had experienced potentially serious pulmonary adverse events) was conducted by expert physicians blinded to the treatment assignment[37]. The reanalysis found no significant difference in pleuritic pain, pneumonitis, or ARDS between these two selected patient subgroups[37]. Such a reanalysis, however, cannot entirely rule out the possibility of an increased risk of pulmonary adverse events secondary to the receipt of pathogen-reduced (compared with non–pathogen-reduced) platelets, as recorded by the initial analysis of the data collected in the trial.

## CAN PR PROTECT FROM ALL PATHOGENS?

Emerging pathogens are unpredictable, yet PR systems are validated against pathogens that are already known. Known culprits would not have predicted vCJD whose prions are free of nucleic acids and thus invulnerable to PR. In the U.K., the subclinical form of vCJD would pose a 1/100,000 risk of contracting a fatal disease for methionine homozygotes surviving 5 years post-transfusion[43]. Such would be the risk if (1) the prevalence of subclinical vCJD in U.K. blood donors was 1/10,000 (in accordance with the estimate presented to the U.K. Spongiform Encephalopathy Advisory Committee in March 2010: 1 positive tonsil among 10,000 examined tonsil specimens[44]), (2) the probability of developing disease in methionine homozygotes (40% of the population or 13 patients) surviving ≥5 years after a transfusion (32 patients[45]) was 23% (three patients with full-blown vCJD[45] out of an estimated 13 methionine homozygotes among the 32 patients[45] surviving ≥5 years post-transfusion), and (3) 40% of subclinically infected Britons had sufficient infectivity in blood to infect recipients, same as in the scrapie experiment in sheep[46]. Based on the relative incidence of clinical disease by the end of 2009 between each European country and the U.K.[47], the prevalence of subclinical vCJD in blood donors would be 1/70,000 in France, 1/150,000 in the Netherlands and in Portugal, 1/250,000 in Spain, and 1/1,000,000 in Italy[43].

Optimally prepared PWBD platelets, with as much of the donor plasma as possible removed and replaced by additive solution if this were considered to be the optimal manufacturing method, should not protect from vCJD. This is because a starting infectivity of 10 infectious doses per milliliter of whole blood corresponds to ≥10 infectious doses per milliliter of plasma after white-blood-cell (WBC) reduction, and thus to ≥100 infectious doses per platelet component containing 10 mL of plasma from any individual donor subclinically infected with vCJD[48]. Therefore, although the platelets themselves appear to be devoid of vCJD infectivity[49], there is adequate infectivity in the residual plasma of both PWBD and single-donor platelets stored in additive solution to infect recipients.

Thus, of two TTIs hitherto emerging in the 21$^{st}$ century, one (WNV infection) would be prevented and one (vCJD) would not be prevented by PR. Although all "classical" transfusion-transmitted *viruses* (HIV, HCV, and HBV) would be prevented by PR[1,11,12,13], it cannot be presumed that PR will protect us from the next major TTI to emerge in the future. Only a reduction in the number of allogeneic-donor exposures (through a combination of conservative transfusion guidelines[1] and avoidance of pooled products such as PWBD platelets) can reduce this risk regardless of the characteristics of the next major transfusion-transmitted pathogen to emerge in the future.

## CAN FEWER DONOR EXPOSURES CONFER OTHER (NONINFECTIOUS) BENEFITS?

When single-donor platelets are compared with PRP WBD platelets in studies diagnosing TRALI by the Canadian consensus criteria[50], and conducted in settings simultaneously transfusing both PWBD and single-donor platelets to the same patients under the same conditions[51,52,53], the risk of TRALI is the same with both types of platelet components[4]. In the passive-surveillance (hemovigilance) studies of Robillard et al.[51] and Eder et al.[52], the risk of TRALI was, respectively, 1/40,452[51] (or 1/279,805[52]) transfused platelet pools vs. 1/46,996[51] (or 1/285,630[52]) transfused single-donor platelets. Gajic et al.[53] presented a nested case-control study within an observational study of 901 consecutive ICU patients (74 cases developing TRALI and 74 controls remaining free of TRALI). Cases received 10 single-donor platelets and six platelet pools; controls received three single-donor platelets and three platelet pools. Thus, the cases received more platelet transfusions than the controls, but the type of platelet concentrate did not differ between the cases and the controls[4]. The equal risk of TRALI with single-donor vs. PWBD platelets found in the three studies[51,52,53] is what would be logically predicted because the higher probability of exposure to 50 mL of plasma from each such alloimmunized donor in a U.S. platelet pool probably carries as much TRALI risk on average as the receipt of 300 mL of plasma from a single donor who may similarly have circulating antibodies directed to the transfusion recipient's WBC antigens.

TRALI may be due to donor WBC antibodies, soluble biologic response mediators accumulating during the storage of cellular blood components, or other still-unidentified agents contained in transfused blood components[54]. The vast majority of donor WBC antibody infusions does not cause TRALI, making it possible that antibodies, albeit present in the majority of the TRALI cases, could be a surrogate for another etiologic factor. Nonetheless, avoidance of donor WBC antibodies is the only measure for TRALI prevention hitherto implemented[55]. Because pregnancy is the usual source of WBC antibodies in donors[56], "male-only" plasma was implemented in the U.S. in September 2007[55]. Data on transfusion-related deaths passively reported to the FDA have shown benefit from this policy[57], which also reduced the number of TRALI cases in the Netherlands by 33%[58]. Nonetheless, observational studies have not consistently shown benefit from "male-only" plasma[59,60,61].

It is expected that, to prevent TRALI, future single-donor platelets will be collected exclusively from male donors or screened female donors (i.e., female donors without a history of pregnancy or shown not to have WBC antibodies)[55]. If donor WBC antibodies cause TRALI[62], and if single-donor platelets collected indiscriminately from both male donors and unscreened female donors (with or without a history of pregnancy and/or WBC antibodies) are associated with the same risk of TRALI as PWBD platelets[4,51,52,53], the future single-donor platelets (to be collected exclusively from male donors or screened female donors) should be associated with a *lower* risk of TRALI than the future platelet pools (which will continue to be collected indiscriminately from both male donors and unscreened female donors).

Even if U.S. PRP WBD platelets are prepared in the future as described under the section on vCJD (with as much of the donor plasma as possible removed and replaced by additive solution), the residual ≤20 mL of donor plasma could suffice to cause TRALI. There have been cases of TRALI attributable to this minor (≤20 mL) plasma contribution from unscreened female donors towards a buffy-coat platelet pool suspended in 10 mL of plasma from a male donor[63].

van Stein et al.[64] conducted a hemovigilance study of TRALI during the 2.5 years before the Netherlands converted to "male-only" plasma. They diagnosed TRALI by the Canadian consensus criteria[50] and also worked up all the implicated components for donor-recipient WBC incompatibility, regardless of the gender of the donor or the plasma content of each component. The authors thus avoided the bias introduced by the selective inclusion of sera from female donors of "plasma-rich" components in working up possible TRALI cases. Because approximately 25% of female donors have WBC antibodies[56], such selection bias necessarily associates TRALI with female donors and "plasma-rich" components explaining, in part, the high prevalence of female donors and "plasma-rich" components among the case reports and small case series of TRALI that preceded the promulgation of the Canadian consensus criteria[1].

TRALI usually develops in multiply transfused patients receiving, simultaneously, RBCs, platelets, and plasma (5.3 units on average in the study of van Stein et al.[64]). The 49 TRALI cases in that study[64] received 129 RBCs, 66 WBD platelet concentrates in platelet pools, and 64 units of plasma. Of 10 deaths associated with TRALI[64], two could have been prevented by "male-only" plasma and one by "male-only" platelets. Seven deaths, however, were associated with European RBCs manufactured by the buffy-coat method. Such "buffy-coat RBCs" are already plasma reduced, containing ≤20 mL of plasma. Efforts to reduce the plasma content of either RBCs or platelets by replacing the donor plasma with additive solutions may thus be unlikely to prevent TRALI (if TRALI is indeed caused by donor WBC antibodies[62] and if unscreened female donors continue to be used as a source of WBD platelets).

If that is the case, instead of trying to prevent TRALI by removing as much plasma as possible from the RBC and platelet components, we should strive to prevent TRALI by removing the exposures to the donors themselves[65]. Multicomponent apheresis[66,67] permits the collection of RBCs and platelets (and/or plasma) from the same donation for transfusion to the same recipient. It can thus replace the whole-blood collections of the 20$^{th}$ century and meet all of a patient's transfusion needs with exposure to fewer donors.

Even before multicomponent apheresis is fully implemented, we can reduce exposures to donors through judicious use of single-donor platelets. The PLADO RCT[39] showed a small, but significant ($p < 0.05$), difference in total transfused platelets between patients receiving half-the-standard dose vs. the standard dose. Because patients receiving half-the-standard dose needed prophylactic platelet transfusion every few days, they could have received half a single-donor concentrate the first time and the other half the next time, thereby significantly reducing the number of exposures to allogeneic donors[1].

## CONCLUSIONS

In summary, at their current stage of development, the PR systems for platelets will increase the risk of mild and moderate (albeit perhaps not severe) bleeding complications and they cannot protect from all pathogens. Moreover, the possibility of an increase in pulmonary adverse events secondary to the receipt of pathogen-reduced platelets (as recorded in the initial analysis of the SPRINT RCT[37]) cannot be entirely ruled out. In contrast to these potential risks of pathogen-reduced platelets, compared with PWBD platelets, single-donor platelets can reduce, by at least twofold, the risk of *all* known and emerging TTIs, as well as the risk of TABS, without incurring *any* risk. The fewer donor exposures secured by the use of single-donor platelets may also reduce the risk of TRALI. Thus, regardless of whether PR is adopted, PWBD platelets should be replaced by single-donor platelets.

Objections to this risk-reduction strategy have been presented from Canada[68] and Germany[69] where a higher risk of bacterial contamination secondary to PWBD (rather than single-donor) platelets (Fig. 1) has not been observed[4] because buffy-coat (rather than PRP) platelets are used. Devine et al.[68] hypothesized that the production of buffy-coat platelets may reduce the risk of TRALI secondary to the associated RBC units manufactured by this same process because “buffy-coat” RBCs are plasma reduced. However, seven of 10 TRALI deaths over 2.5 years in the Netherlands[64] were associated with precisely such “plasma-reduced” buffy-coat RBCs and the mechanism of TRALI secondary to RBC transfusion may differ from the antibody-mediated mechanism postulated for the “plasma-rich” components[70]. Schrezenmeier and Seifried[69] proposed that since all platelet transfusion needs of patients not alloimmunized to human leukocyte antigens can be met by pooling already-available buffy coats, any donor reactions to plateletapheresis collections represent an “additional” and, thus, hard-to-justify risk. In the future, however, multicomponent apheresis can meet all transfusion needs of U.S. patients by subjecting a number of donors to the rare risks of donation that will not exceed the number of donors bled today to produce the same number of components from whole-blood donations[71]. Furthermore, donors who self-select for plateletapheresis (as well as plasmapheresis or “double” RBC apheresis) donations have fewer ($p < 0.05$) reactions than whole-blood donors[72].

The evidence published through June 30, 2010 thus precludes the use of PWBD platelets in any risk-reduction strategy. Our choices are limited to non–pathogen-reduced vs. pathogen-reduced single-donor platelets. The drawback of PR is the increase in the risk of mild and moderate (and potentially also severe, Fig. 3) bleeding complications, as well as perhaps an increase in the risk of pulmonary adverse events[37]. Observational studies after the implementation of PR in some countries[73,74] did not record such increased bleeding among the recipients of pathogen-reduced platelets, but neither did they document the expected increase in the number of platelet transfusions owing to the platelet losses caused by PR. Most likely, platelet transfusions had been previously overutilized, so that the effective reduction in platelet dose caused by PR did not result in a visibly increased need for platelets. It is also possible that an increase in mild and moderate bleeding complications might have gone unnoticed outside the framework of an RCT.

The immediate benefit from PR would be the prevention of perhaps as many as 90% of cases of TABS associated with single-donor platelets[75,76], excepting TABS secondary to some spore-forming bacteria. TABS continues to represent a risk estimated at 1/60,000 non–pathogen-reduced (but bacterially screened) single-donor platelets[1], assuming a 50% reduction of the historical risk of TABS[6] thanks to process improvements (donor selection, better arm cleansing, and/or diversion of the first aliquot of blood most likely to contain the skin contaminants[75]), and a further 50% reduction thanks to bacterial culture[77]. TABS is usually not detected, however, because neutropenic hemato-oncology patients often succumb to infection without the platelet transfusion being considered as the potential source of the infection. Although TABS was reported to occur before 2004 with a frequency between 1/3,000[6] and 1/100,000[78] platelet transfusions, figures toward the lower end of that range cannot be reconciled with the risk estimates produced by the more recent hemovigilance reports[79] (1/75,000) following implementation of all the aforementioned measures (including bacterial culture). Thus, because the risk of TABS rises with bacterial proliferation during storage, the storage period of platelets (5 days in the U.S.) was reduced to 4 days in Germany and to 3 days in Japan[80].

The question that cannot be answered from the available data is whether the reduced capacity of pathogen-reduced single-donor platelets to prevent bleeding complications in recipients of pathogen-reduced (compared with non–pathogen-reduced) platelets is due only to the loss of a proportion of the pathogen-reduced platelets or to functional impairment of all the pathogen-reduced platelets as well. Thus, the research question continues to be whether pathogen-reduced single-donor platelets are capable of preventing bleeding and investigations of the cost effectiveness of PR (such as whether PR can permit extension of the allowed storage period of platelets[42,81]) are premature.

Further research and development are needed vis-à-vis the PR of platelets and the efficacy and safety of the pathogen-reduced components has to be demonstrated in future RCTs (as discussed publicly at the November 2009 meeting of the FDA's Blood Products Advisory Committee[82]). It is hoped that transfusion of pathogen-reduced single-donor platelets will eventually emerge as the optimal risk-reduction strategy for TABS and TTIs if these technologies are licensed by the FDA[1]. To choose between pathogen-reduced and non–pathogen-reduced single-donor platelets, the increased risks of bleeding (and perhaps also pulmonary) complications, as well as the uncertainty about the nature of the platelet damage caused by PR, need to be, respectively, precisely quantified and elucidated, and then weighed against the competing risks of TABS and emerging TTIs.
